# South African speech-language therapists’ and audiologists’ professional quality of life

**DOI:** 10.4102/sajcd.v71i1.1042

**Published:** 2024-08-23

**Authors:** Victor M. de Andrade, Cherilyn da Silva, Nicky Israel

**Affiliations:** 1 Department of Speech Pathology and Audiology, Faculty of Humanities, University of the Witwatersrand, Johannesburg, South Africa; 2 Department of Psychology, Faculty of Humanities, University of the Witwatersrand, Johannesburg, South Africa

**Keywords:** professional quality of life, speech-language therapists, audiologists, compassion satisfaction, burnout, secondary traumatic stress, South Africa, compassion fatigue

## Abstract

**Background:**

Limited research is available regarding the professional quality of life experiences of South African speech-language therapists and audiologists, despite the implications this has for wellbeing, quality of patient care, productivity and attrition from the professions.

**Objectives:**

This study explored levels of compassion satisfaction, burnout, and secondary traumatic stress, the relationships between these, differences on the basis of registration and years of experience and participants’ perceptions of their professional quality of life.

**Method:**

A sample of 92 South African speech-language therapists and audiologists completed an online survey that included the Professional Quality of Life (ProQOL) scale. The data were analysed using descriptive statistics, analysis of variation (ANOVA), correlations and thematic analysis.

**Results:**

The findings indicated that participants experienced slightly higher levels of secondary traumatic stress and burnout and slightly lower levels of compassion satisfaction than international samples. There were significant inter-relationships between the three elements of professional quality of life, and no significant differences for these on the basis of registration or years of experience. Participants identified a range of factors that contributed to their experiences of compassion satisfaction and fatigue, as well as suggestions for improvement.

**Conclusion:**

Professional quality of life plays an important role in South African speech-language therapists and audiologists’ professionalism, job performance and satisfaction and retention.

**Contribution:**

The data collected provide valuable insights into the professional quality of life experiences of South African speech-language therapists and audiologists, as well as those working in similar contexts. It also offers suggestions that may contribute to future research and interventions.

## Introduction

Professional quality of life represents peoples’ lived experience at work and their subjective judgement about how satisfied they are with this experience (Barofsky, 2012; Ravi et al., 2016). In the helping professions, it is typically assessed in terms of the secondary effects of helping others (De La Rosa et al., 2018; Garner & Golijani-Moghaddam, 2021; Stamm, 2010). Compassion satisfaction refers to pleasant feelings, such as joy, pleasure, gratification and invigoration derived from helping others and doing one’s job well (Stamm, 2010; Sukut et al., 2022). Secondary traumatic stress is characterised by negative feelings and behaviours that result from persistent exposure to clients’ traumas, including fear, depression, stress, memory lapses, avoidant behaviour and intrusive thoughts (Stamm, 2010; Sukut et al., 2022). Burnout encompasses levels of emotional exhaustion, frustration, anger, depersonalisation and reduced personal accomplishment resulting from work-related stressors (Ravi et al., 2016; Stamm, 2010; Sukut et al., 2022). Together, secondary traumatic stress and burnout are often seen as constituting compassion fatigue, a phenomenon that occurs when professionals feel less able to provide care and empathy to others or to manage others’ suffering (Cavanagh et al., 2020; Ravi et al., 2016).

Professional quality of life varies across countries because of differences in healthcare systems, professional practices, education, individual and workplace culture and economic circumstances (Brännström et al., 2016; Gandi et al., 2011; Ravi et al., 2016). In South Africa, healthcare professionals, including speech-language therapists and audiologists (SLT&As), face numerous challenges including burden of disease, infrastructural and accessibility constraints, skills and resource shortages, demand-supply mismatches, implementation issues and linguistic, cultural and economic barriers (Harris et al., 2011; Maphumalo & Bhengu, 2019). These are compounded by contextual factors such as traumatic stress because of experiences of crime and violence and the effects of poverty (Burger & Christian, 2020; de Andrade, 2015; De La Porte & Davids, 2016). The impact of the South African socio-economic and politico-cultural context can also be seen in the strain on available healthcare, large caseloads, time and resource constraints and the geographical distribution and demographics of qualified SLT&As (Penberthy et al., 2018; Pillay et al., 2020; Swidler & Ross, 1993). There is also an estimated supply-need gap of approximately 2800 SLT&As (Pillay, 2020). These challenges pose significant risks to professional quality of life, with SLT&As reporting experiencing guilt, helplessness, hopelessness, workplace anxiety and professional avoidance in their practice in South Africa (Nagdee & de Andrade, 2023).

There is minimal research available that has directly explored professional quality of life in South African SLT&As. Swidler and Ross (1993) identified moderate levels of emotional exhaustion, low levels of depersonalisation and high levels of personal accomplishment among South African SLT&As at that point in time, while Nagdee and de Andrade (2023) found that South African SLT&As experienced emotional exhaustion, despondence and guilt as a result of working with terminal patients. Although other studies (e.g., Brito-Marcelino et al., 2020; Giddens et al., 2022) have included South Africans as part of their sample, there appears to be no additional work that has directly explored the secondary effects of treating patients on SLT&As in the South African context.

Professional quality of life has implications for practitioners’ wellbeing, quality of patient care, productivity and attrition from the professions and is especially important to consider in South Africa where SLT&As work with the aforementioned challenges (Albott et al., 2020; McLaughlin et al., 2008; Ravi et al., 2016; Swidler & Ross, 1993). The interplay between various aspects of work and the context in which the profession is practised, together with personal and professional demands, adds to the complexity of professional quality of life among South African SLT&As.

Given this and the need for more research, the aim of the current study was to gain insight into reported professional quality of life in South African SLT&As. Specific objectives included: measuring professional quality of life and comparing these scores between different professional registration types and years of practice, establishing the nature of the relationships between compassion satisfaction, burnout and secondary traumatic stress in the sample and exploring SLT&A’s reported personal reflections and experiences of their professional quality of life and how this could be improved.

## Research methods and design

The study was cross-sectional and survey-based; both quantitative and qualitative data were collected using an online questionnaire (Jhangiani et al., 2019). The questionnaire included demographic questions regarding occupation, years of experience and work setting; a slightly modified version of the Professional Quality of Life (ProQOL) scale version 5 (Stamm, 2010) and four open-ended questions regarding emotions experienced by the participants in relation to compassion in their work, possible improvements for quality of professional life and other comments they wished to include. The questionnaire was hosted on Research Electronic Data Capture Software (REDCap^©^).

The ProQOL scale version 5 is a 30-item, self-report measure that gives an indication of the frequency of behaviours, emotions and/or thoughts associated with compassion satisfaction (10 items), burnout (10 items) and secondary traumatic stress (10 items) experienced over the last 30 days by the respondent (Stamm, 2010). Each item is answered on a 5-point, Likert-type scale ranging from ‘never’ to ‘very often’ and higher scores indicate a higher level of the construct being assessed. The ProQOL scale version 5 is not a formal diagnostic tool but can give a preliminary indication of possible burnout or secondary traumatic stress using pre-established cut-off scores (Stamm, 2010). Stamm (2010) notes that the scale has good internal consistency reliability, with estimates ranging between 0.75 and 0.88 for the different subscales, good construct validity and a wide range of use in different professional contexts. In the study, permission to use and slightly modify the ProQOL scale version 5 wording was granted; these modifications replaced the generic ‘help’ and ‘helper’ terms in the original measure with study-specific phrases such as ‘speech-language pathologist and/or audiologist’, ‘provide services to’ and ‘people I help at work’.

The population of interest was professional speech-language therapists, audiologists and dually qualified SLT&As registered with the Health Professions Council in South Africa (HPCSA); aside from professional qualification, there were no other exclusion criteria for the sample. In South Africa, the entry requirement to register to practise is the completion of a field-specific or dual 4-year bachelor degree (Honours-equivalent); dual registration and practise are common because of historical, conceptual and practical relations between the two professions (Pillay, 2020). The sampling strategy was non-probability, convenience and snowball sampling, and participation was voluntary (Jhangiani et al., 2019). The South African Speech-Language-Hearing Association; the South African Association of Audiologists; the National Black Speech, Language, and Hearing Association and Rural Rehabilitation South Africa agreed to disseminate an e-mail to their members containing information about the study, a distress protocol and a secure link to the online questionnaire, and those contacted were asked to forward the email to any other potential participants they knew of who might be interested in taking part.

The final sample for the current study consisted of 92 participants, with 23 (25.0%) registered audiologists, 48 (52.2%) registered speech-language therapists, and 21 (22.8%) dually registered SLT&As. For work experience, 28 (30.4%) participants had been practising for between 1 and 5 years; 33 (35.9%) had been practising for between 6 and 15 years and 31 (33.7%) had been practising for 16 years or more. A large majority of the sample worked in urban (*n* = 66 [71.7%]) or both urban and rural (*n* = 16 [17.4%]) environments; 10 (10.9%) participants worked only in a rural environment, and 48 (52.2%) participants practised in more than one location. Furthermore, 59 (64.1%) participants reported working in private practice; 40 (43.5%) in public hospitals; 30 (32.6%) in schools; 10 (10.9%) in a university setting on a full-time basis; 11 (12.0%) in a university setting on a part-time basis; 3 (3.3%) for a professional board and 5 (5.4%) in an alternate work site.

Potential participants were provided with a participant information sheet and were informed that completion of the questionnaire would be taken as informed consent to participate in the study. The data collected was anonymous and was stored securely. After completion of the questionnaire, each participant was provided with their individual ProQOL scores, general score interpretations, a distress protocol and a list of referral options and resources.

Statistical analysis of the quantitative data was carried out using IBM SPSS (Statistical Package for the Social Sciences) Statistics^©^ version 28 (IBM Corp., 2021). Cronbach Alpha coefficients were calculated to check the internal consistency reliability of the ProQOL scores in the sample (Field, 2009). Descriptive statistics were used to summarise participants’ occupation, years of experience, work setting, and ProQOL scores and to check normality of the data. One-way analysis of variance (ANOVA) was used to compare ProQOL scores between the different professional registration and years of practice groups; and Pearson’s correlation coefficients were calculated to represent the relationships between the ProQOL subscales (Field, 2009). There were no adjustments to the data, and assumptions for all statistical techniques were sufficiently met (Field, 2009). Data from the open-ended questions were analysed using thematic analysis (Braun & Clarke, 2006; Knudsen et al., 2012). Steps in the process included familiarisation, selection, categorisation, coding and interpretation, and the reliability of the analysis was informed by the consistency of the data coding and how well it was supported by triangulation (Braun & Clarke, 2006; Knudsen et al., 2012).

### Ethical considerations

Ethical approval to conduct this study was obtained from the University of the Witwatersrand Department of Speech Pathology and Audiology Human Research Ethics Committee (reference number: STA_2019_21).

## Results

The Cronbach Alpha estimates for the ProQOL subscales were α = 0.90 for compassion satisfaction, α = 0.81 for burnout and α = 0.86 for secondary traumatic stress, indicating high internal consistency reliability for the scale in the sample (Field, 2009). The standard cut-off scores indicated that no participants reported high levels of burnout and only 1 (1.1%) participant reported high levels of secondary traumatic stress; while 31 (33.7%) participants reported high levels of compassion satisfaction. Table 1 presents the frequencies for the cut-off scores, the means, standard deviations, minimum and maximum scores and skewness and kurtosis estimates for the ProQOL subscales. All of the ProQOL subscales were normally distributed in the sample, with a majority of participants reporting average levels of compassion satisfaction, burnout and secondary traumatic stress.

**TABLE 1:** Descriptive statistics for the Professional Quality of Life (ProQOL) subscales.

Subscales	Mean	s.d.	Min.	Max.	Skewness	Kurtosis	Low		Average		High	
			*n*	*n*			*n*	%	*n*	%	*n*	%
Compassion satisfaction	38.61	6.189	22	50	−0.337	0.116	1	1.1	60	65.2	31	33.7
Burnout	25.76	6.135	14	38	0.041	−0.747	30	32.6	62	67.4	-	-
Secondary traumatic stress	25.41	7.034	12	42	0.330	−0.486	35	38.0	56	60.9	1	1.1

Max., maximum score; Min., minimum score; s.d., standard deviation.

Comparison of the different occupation groups indicated that there were no significant differences in levels of compassion satisfaction (*F*_2;89_ = 0.348; *p* > 0.05; η^2^ = 0.008); levels of burnout (*F*_2;89_ = 1.639; *p* > 0.05; η^2^ = 0.036) or levels of secondary traumatic stress (*F*_2;89_ = 1.624; *p* > 0.05; η^2^ = 0.035) between those participants who were registered as speech-language therapists, those who were registered as audiologists and those who had a dual registration. Unexpectedly, comparison of the different experience groups also indicated that there were no significant differences in levels of compassion satisfaction (*F*_2;89_ = 0.299; *p* > 0.05; η^2^ = 0.007); levels of burnout (*F*_2;89_ = 1.865; *p* > 0.05; η^2^ = 0.040) or levels of secondary traumatic stress (*F*_2;89_ = 1.562; *p* > 0.05; η^2^ = 0.034) between those participants who had been practising for between 1 and 5 years, those who had been practising for between 6 and 15 years and those who had been practising for 16 or more years. Homogeneity of variance between the groups was established for all comparisons, and, in all cases, the effect sizes were small (Field, 2009).

Table 2 presents the inter-correlations between the ProQOL subscales. There was a significant, negative and strong relationship between compassion satisfaction and burnout (*r* = −0.731; *p* < 0.001), while the relationship between compassion satisfaction and secondary traumatic stress (*r* = −0.326; *p* < 0.01) was significant, negative and moderate. Burnout and secondary traumatic stress (*r* = 0.657; *p* < 0.001) were significantly, positively and strongly related to one another (Field, 2009).

**TABLE 2:** Inter-correlations between the Professional Quality of Life (ProQOL) subscales.

Subscales	Pearson’s *r*	*p*	Lower CI	Upper CI
Compassion satisfaction and burnout	−0.731	< 0.001	−0.814	−0.618
Compassion satisfaction and secondary traumatic stress	−0.326	0.002	−0.497	−0.129
Burnout and secondary traumatic stress	0.657	< 0.001	0.522	0.759

CI, confidence interval.

Four themes emerged from the thematic analysis: (1) satisfaction, (2) dissatisfaction with working conditions, (3) the emotional impact of work and (4) suggestions for improvement.

The first theme was satisfaction, which was associated with providing effective and high-quality diagnosis, treatment and guidance to patients and their families; helping patients and families to gain confidence and skills and achieve their goals; seeing patient improvement and/or growth and helping those with limited resources:

‘I love what I do and I love seeing patients improve and be rehabilitated and able to return to work and their lives … [*to*] know that in some way I helped them achieve that result is indescribable.’ (Participant 32, dual SLT&A, 6–15 years ‘experience)

‘The tears rolling down a mothers [*sic*] face when you give their child a hearing aid and they start repeating what you are saying. Nothing beats that feeling.’ (Participant 54, audiologist, 1–5 years’ experience)

Circumstances that led to cognitive appraisals of success, such as receiving gratitude or feeling that one was making a difference or fulfilling one’s life purpose, were also associated with feelings of professional accomplishment, appreciation, recognition and satisfaction:

‘When a patient thanks you for “changing their lives”, it is one of the most rewarding feelings …’ (Participant 79, speech-language therapist [*SLT*], 1–5 years’ experience)

‘Each time my clients make progress or achieve something in therapy or make a joke in a session I’m reminded about how happy my job makes me. I love seeing kids enjoy learning and achieve success. My job is God’s gift to me.’ (Participant 21, SLT, 1–5 years’ experience)

Feeling satisfied was closely associated with forming meaningful connections and building relationships with patients and their families and being able to offer them empathy and emotional support, as well as seeing their positive reactions to any progress made:

‘Working closely with a child and their family to improve … and seeing the progress and joy it brings in that child and that family’s lives …’ (Participant 37, SLT, 6–15 years’ experience).

Manageable caseloads, access to resources, sufficient time to prepare and interact with patients, opportunities for professional growth, being able to work as part of a team, having positive relationships with colleagues and novelty in the work environment were identified as factors that could contribute to success and thus satisfaction. Many of these mirrored the concerns raised by participants in relation to working conditions and the emotional impact of their work.

The second theme was dissatisfaction with working conditions, which included concerns about high patient caseloads, staff and resource shortages, lack of access to treatment and rehabilitative services, restrictive workplace policies and regulations, lack of oversight and broader systemic and social issues such as poverty and unemployment. These challenges were associated with feelings such as frustration, anger and inadequacy because of an inability to meet patient needs, provide quality services or manage personal workload effectively. As reflected in the following quotes, some participants expressed feelings of emotional exhaustion, disconnection and futility resulting from working in a system perceived to be both inadequate and non-responsive:

‘[*C*]aseloads are very high, mostly because we struggle to fill vacant … posts … Having to split yourself in a million pieces whilst fighting to still be the best you can be for each person without compromise. Juggling all the balls in the air and at the end of the day … you are drained physically and emotionally, and it wasn’t one thing, it was the tornado of needs [*physical and sometimes emotional*] that had to be met …’ (Participant 29, SLT, 6–15 years’ experience)

‘The workload, feeling that what I do doesn’t really make a difference because it’s not enough and because other systems are not in place to support patients …’ (Participant 88, SLT, 6–15 years’ experience)

Other work-related concerns raised by participants included financial stress, administrative demands, lack of opportunities for employment and professional growth, role expectations and the intensity of the work. Participants also expressed anger, sadness and frustration as a result of interpersonal conflict, difficult interactions with patients or relatives and a perceived lack of recognition, acknowledgement or support from other professionals or management. As reflected in the following quote, the lack of validation from others in the system created feelings of ineffectuality, despondency and low valuation:

‘I find drs [*sic*] ignoring my recommendations … and discharging pts [*sic*] without acknowledging the critical role I play to be very disheartening and depressing. Especially after I have advocated on behalf of the [*patient*] … I am still dismissed and my pt [*sic*] still dies or suffers a terrible fate that I know is preventable if they just listened to me. I feel angry, and very sad for the patient.’ (Participant 28, SLT, 6–15 years ’ experience)

The third theme, which was closely connected with the emotional consequences of the sources of dissatisfaction in the second theme, reflected the emotional impact of the work carried out by SLT&As. This included concerns about constant exposure to trauma, over-involvement with patients, and the effects of being emotionally available and acting as a source of support, as well as feelings of helplessness, inadequacy, guilt, and sadness if patients or their families needed to be informed about or had a poor or terminal diagnosis or prognosis:

‘Extreme sadness in what some patients go through daily or have been through, when they confide in you.’ (Participant 91, audiologist, 6–15 years’ experience)

‘Sometimes no matter how you try you can’t help someone improve and patients can even die. That’s the hardest part …’ (Participant 10, SLT, 1–5 years’ experience)

Participants also raised concerns about maintaining professional boundaries and struggling to separate their personal and professional life. This was associated with a lack of self-care as well as reported feelings of emptiness, exhaustion, burnout, desensitisation, sadness and helplessness. Some participants expressed that they felt unable to offer additional support to others in their personal life or to perceive their own or loved ones’ problems as important because of their experiences at work:

‘I often struggle to keep my work-related-worries about the clients restricted to office hours. I always take my work and work-related-worries back home which affect my personal life and sleep.’ (Participant 18, SLT, 6–15 years ’ experience)

‘I often feel that I am less compassionate to family and friends in need as my ‘tank’ is empty …’ (Participant 14, SLT, 16 or more years’ experience)

Participants identified disengagement and depersonalisation as coping mechanisms for work situations that evoked feelings of being overloaded, overly committed, unappreciated or resentful. A desire for more emotional support was also expressed:

‘It feels like the only way to get through it is to be a robot and not care or feel, even though as human beings that is what we inherently do.’ (Participant 63, SLT, 6–15 years’ experience)

The final theme was suggestions for improvement, many of which directly reflected proposed resolutions to the work challenges and emotional impact of the work concerns raised by participants. These included: improved SLT&A-patient ratios and reduced caseloads; clearer guidance for referrals and scope of intervention; increased access to resources supporting effective practice; reduced bureaucracy; increased pay and compensation and administrative assistance. Participants also recommended growing the profession and improving the healthcare system generally; increasing the number of government posts for SLT&As and improving patient access to treatment and options for referral.

There was a strong emphasis on the need for holidays, time-outs, flexible working hours and opportunities for self-care as a means of ensuring better mental health. Work–life balance, boundaries between work and home and involvement in activities outside of work were strongly advocated for:

‘Professionals should learn to take time for themselves and spent [*sic*] it away from the work situation. Activities like postgraduate studies, hobbies and alone time is [*sic*] very important …’ (Participant 25, SLT, 16 or more years’ experience)

One of the strongest themes that emerged was the need for mandatory and accessible psychological supervision and opportunities for formal debriefing, reflection and obtaining emotional support. Informal support through debriefing or venting to colleagues, building support networks with other SLT&As, mentoring and collaboration and enhanced collegiate relationships and teamwork were also advocated for, as were increased recognition and support from management and other professionals:

‘Regular mental health checks that are a compulsory part of the job … the time and opportunity within a work day to process the trauma of what we have to manage. It is not normal to have to see the violence of the gang infested areas on a daily basis and the impacts thereof, knowing your hands are tied in trying to help the community where it matters. It’s not normal to have to treat young children whose lives have been altered significantly because of careless violence. It’s not normal to be expected to continually see and treat palliative patients without the needed emotional/debriefing support. All of this needs to be recognised by those who are in management positions …’ (Participant 63, SLT, 6–15 years’ experience)

‘… I strongly believe we should be a “supervised” profession in the way that social workers and psychologists see a mental health professional regularly to have debriefing and look after one’s own emotional health and well-being.’ (Participant 77, audiologist, 16 or more years’ experience)

Participants felt that improving public and professional awareness of the role of SLT&As as an allied health profession and the profile of SLT&A work in South Africa would be useful to encourage early referral and external valuation. More opportunities for training and improved knowledge and skills, as well as access to a greater range of affordable Continued Professional Development (CPD) activities, were also recommended as ways in which to promote successful intervention and treatment. Job satisfaction, a positive mindset, opportunities for novelty, finding a good work-environment fit and ensuring a good personality fit with the profession were also mentioned:

‘Awareness of what audiologists and speech therapists do professionally, so we can be sought out by those who need our services. Also, a greater awareness by other professionals, particularly doctors, as to how we can benefit patients as part of a team …’ (Participant 47, dual SLT&A, 16 or more years’ experience)

## Discussion

Professional quality of life represents both the positive and negative emotional and psychological effects that occur as a result of experiences in the work environment (Stamm, 2010). For healthcare professionals, the nature of their work with patients may lead to a particular vulnerability to compassion fatigue, represented by both secondary traumatic stress and burnout, with negative consequences for physical and mental health as well as impaired clinical judgement and behaviour (Cocker & Joss, 2016; Gustaffson et al., 2022; Stamm, 2010; Zimmer et al., 2022). Positive experiences of compassion satisfaction can mitigate against these effects and can potentially increase sensitivity and empathy and improve the quality of practitioner–patient relationships (Ryu & Shim, 2022; Stamm, 2010). In both instances, the emotional consequences of caring represented by professional quality of life can have implications for job performance, quality of patient care, professionalism, job satisfaction, retention and turnover and sustainability (Albott et al., 2020; Jensen et al., 2022; Ravi et al., 2016). Despite its importance for all healthcare workers, there is comparatively limited research that has explored professional quality of life for speech-language therapists and audiologists. There is also almost no research available that has examined this in South African SLT&As, despite the potential utility of this information and the role played by context and individual circumstances in shaping these experiences (Barofsky, 2012; Ravi et al., 2016; Stamm, 2010).

To address this gap, the current study explored the reported professional quality of life in a sample of South African SLT&As using a survey-based methodology. This included measuring compassion satisfaction, burnout and secondary traumatic stress experienced by participants and exploring the relationships between these, as well as comparing these for different professional registrations and years of practice. Participants’ perceptions and experiences related to their professional quality of life and how this could be improved were also explored through thematic analysis of their responses to open-ended questions in the survey.

In the current study, 33.7% of the participants reported high levels of compassion satisfaction, 65.2% reported average levels of compassion satisfaction and 1.1% reported a low level of compassion satisfaction. This pattern is an interesting variation from similar profiles identified in international samples. For example, Giddens et al. (2022) found that 45.8% of their sample of audiologists from eight countries reported high levels of compassion satisfaction and 54.2% reported moderate levels; while Ravi et al. (2016) found that 48.0% of their sample of SLT&As working in India reported high levels of compassion satisfaction and 51.6% reported average levels. Zimmer et al. (2022) found that 64.0% of their sample of audiologists working in the United States reported high levels of compassion satisfaction and 36.0% reported moderate levels, while Severn et al. (2012) found that 25.0% of their sample of New Zealand audiologists had high levels of compassion satisfaction, and 22.0% reported low levels of compassion satisfaction. These patterns may reflect differences in context, sample composition and individual participant characteristics but also indicate that levels of reported compassion satisfaction were slightly lower for South African SLT&As in the sample relative to international trends. Similarly, for secondary traumatic stress, 38.0% of the participants in the study reported low levels of secondary traumatic stress, 60.9% reported average levels of secondary traumatic stress, and 1.1% reported a high level of secondary traumatic stress. Zimmer et al. (2022) found that 83.0% of their participants reported low secondary traumatic stress and 17.0% reported moderate levels of traumatic stress, while Ravi et al. (2016) found that 54.0% of their sample reported low levels of secondary traumatic stress and 46.5% reported moderate levels of secondary traumatic stress. Levels of secondary traumatic stress thus seemed to be slightly higher in the current study relative to international findings. These results may reflect the emotional consequences of common challenges experienced by South African SLT&As, which include staff shortages; demand-supply mismatches; limited access to infrastructure and resources necessary to offer effective treatment and continual exposure to the effects of poverty, violence and deprivation (Burger & Christian, 2020; De La Porte & Davids, 2016; Harris et al., 2011; Maphumalo & Bhengu, 2019).

There also seemed to be slightly higher levels of burnout in the study compared to trends observed in international samples. In the current study, 32.6% of the participants reported low levels of burnout and 67.4% reported average levels of burnout. Ravi et al. (2016) found that 60.6% of their participants reported low levels of burnout and 39.4% reported average levels of burnout, while Giddens et al. (2022) found that 55.9% of their participants reported low levels of burnout and 44.1% reported average levels of burnout. Zimmer et al. (2022) found that 61.0% of their sample reported low levels of burnout and 39.0% reported moderate levels of burnout. These patterns could reflect variations in the job demands, job roles and expectations of SLT&As across different countries as well as the psychological effects of the situational challenges common in South Africa (HPCSA, 2009; 2012; Maphumalo & Bhengu, 2019; Ravi et al., 2016).

There was a significant, strong and positive relationship between burnout and secondary traumatic stress in the sample, which aligns with both existing theory and previous research findings (cf. Geoffrion et al., 2019; Ravi et al., 2016; Severn et al., 2012; Stamm, 2010; Zimmer et al., 2022). As anticipated, compassion satisfaction was also significantly and negatively correlated with both burnout and secondary traumatic stress (cf. Geoffrion et al., 2019; Giddens et al., 2022; Stamm, 2010; Zimmer et al., 2022). It was, however, interesting to note that there was a weaker relationship between compassion satisfaction and secondary traumatic stress than between compassion satisfaction and burnout. This suggests that the positive emotional impact of their work experienced by South African SLT&As may be associated less with their exposure to patient trauma than with their experiences of the work environment as supportive or not and associated feelings of exhaustion and disengagement (Shoji et al., 2015; Siegfried, 2008; Stamm, 2010). This was echoed in the themes that emerged from the qualitative data regarding dissatisfaction with working conditions and the emotional impact of the work and has implications for potential intervention. Further research is warranted to gain a more nuanced understanding.

Regarding occupation, there were no significant differences in the levels of compassion satisfaction, burnout or secondary traumatic stress experienced by those participants registered as speech-language therapists, those registered as audiologists or those with a dual registration in the sample. This was anticipated to an extent given the similarities in work roles and expectations between the registrations (HPCSA, 2009, 2012) but did contradict the finding by Ravi et al. (2016) of a significant difference in levels of secondary traumatic stress between speech-language therapists and audiologists. Ravi et al. (2016) attributed this difference to variations in age, work demands, and work settings; in South Africa, the general shortage of SLT&As, high patient load, cross-setting work and shared context may account for the similarities in scores observed (Goswami et al., 2018; Pillay et al., 2020).

There were also no significant differences in levels of compassion satisfaction, secondary traumatic stress or burnout based on years of experience in the current study. This was slightly surprising as fewer years of experience is often considered a risk factor for increased levels of burnout, compassion fatigue or occupational stress (Marante & Farquharson, 2021; Sinclair et al., 2017; Swidler & Ross, 1993) and a few studies (cf. Blood et al., 2008; Ravi et al., 2016) have found higher levels of burnout for those with less experience in SLT&A samples. There are, however, also a number of studies that have identified no relationship between years of experience and burnout or occupational stress in SLT&A samples (cf. Brännström et al., 2016; Emanuel, 2021; Giddens et al., 2022; Severn et al., 2012), and Cavanagh et al. (2020) argue that the empirical association between compassion fatigue and years of experience is somewhat unclear. It has been proposed that less experienced practitioners may experience more burnout or occupational stress as a result of financial uncertainty, role overload, less experiential knowledge and a perceived lack of control; while more experienced practitioners may experience these because of increased managerial demands, expanded work roles, changes to the profession and shifts in technology, which may account for similar levels of reported burnout across different ranges of experience (Brännström et al., 2016; Giddens et al., 2022; Ravi et al., 2016; Severn et al., 2012). There is, however, a need for further research to clarify the nature of the association between indicators of professional quality of life and years of experience for SLT&As, as well as the role played by age in relation to these (Zimmer et al., 2022).

There is considerable research available that has explored work-related and environmental factors contributing to occupational stress and professional quality of life in SLT&As (cf. for example, Brännström et al., 2016; Brito-Marcelino et al., 2020; Ewen et al., 2021; Giddens et al., 2022; Kumar et al., 2022; Ravi et al., 2016; Severn et al., 2012; Zimmer et al., 2022). These include time demands, work–life balance, staff shortages, finances, equipment and technology, administration, practice setting, bureaucratic restrictions, management, job autonomy, professional devaluation, workplace politics, access to professional support, training and professional development, the nature of the work and treatment demands, patient contact and expectations, patient accountability and individual traits (Brännström et al., 2016; Brito-Marcelino et al., 2020; Ewen et al., 2021; Giddens et al., 2022; Kumar et al., 2022; Ravi et al., 2016; Severn et al., 2012; Zimmer et al., 2022). While the themes raised in the qualitative data collected in the study essentially reflect these previously identified factors, they also highlight unique connections between these and experiences of compassion satisfaction and compassion fatigue for South African SLT&As.

There were four broad factors that participants in the study identified as contributing to their experiences of compassion satisfaction through eliciting feelings of contentment, gratification and achievement. These were providing high-quality and effective care for patients, seeing patient improvement and/or growth, receiving acknowledgement or gratitude for efforts made and having a positive work environment and work experience. These factors mirrored many of the specific concerns raised by participants, and this further highlights the importance of considering these for potential targeted workplace interventions to create positive work-related emotional experiences for South African SLT&As.

There were two distinct sets of factors that participants associated with experiences of compassion fatigue – one set related to working conditions and the other to the emotional impact of the SLT&A role. For working conditions, participants highlighted challenges such as high caseloads, staff and resource shortages, excessive bureaucracy and access restrictions, financial stress, administrative demands, limited opportunities for growth, interpersonal conflict and workplace politics, insufficient professional support and negative patient interactions. These were presented as situational factors that elicited feelings of frustration, anger, sadness, inadequacy and being overwhelmed in participants. Participants also expressed feelings of helplessness, guilt, sadness, desensitisation, exhaustion, resentment and a desire to disengage as responses to inherent but emotionally demanding elements of their work as SLT&As. These included repeated exposure to trauma, supporting and counselling patients and their families, delivering poor or terminal prognoses, delineating and maintaining personal and professional boundaries and being emotionally available outside of the work environment. Taken together, these challenges form an extensive list of situational and work-inherent factors that may lead to the development of burnout or secondary traumatic stress in South African SLT&As. The similarity between these and factors identified internationally supports the transferability and implementation of existing intervention strategies designed to reduce compassion fatigue in the South African SLT&A population but also highlights the value of exploring which unique combinations of factors play a stronger determinative role in any particular situation to support contextual adaptations and thus achieve the highest efficacy for these.

The suggestions for improving professional quality of life raised by participants were also varied and clearly reflective of individual experience but included several common threads that could form an important base for further research and intervention. The provision of formal and accessible psychological support and supervision, formation of peer support networks, enabling opportunities for self-care and time away from work and assistance in maintaining boundaries and a successful work–life balance were advocated for particularly strongly by participants. These align closely with existing understandings of the emotionally demanding nature of the work carried out by SLT&As and represent important strategies for maintaining mental health for this group of professionals both in South Africa and generally (Giddens et al., 2022; HPCSA, 2009; 2012; Zimmer et al., 2022). Various ways in which to improve working conditions, job and training opportunities and the public profile of the work carried out by SLT&As were also recommended by participants. Some of these recommendations were beyond the level of the individual employee and would require support and investment from management and government, for example, more posts, additional paid vacation leave, and extra funding. There were, however, other recommendations that could be implemented through increased training and changes to professional culture designed to foster a healthy work-life balance, engagement in self-care, reduced workplace anxiety and debriefing (Nagdee & de Andrade, 2023). Further research to identify ways in which to achieve these outcomes in a resource-effective manner within existing systems of practice is thus warranted.

Although this study provided valuable insights into experiences of professional quality of life and how this could be improved for both South African SLT&As and SLT&As generally, there were also a number of important limitations. One of these was the restricted amount of demographic information that was collected from participants; additional data for factors such as age, socioeconomic status, relationship status, living conditions, workload and perceived job control and workplace support could have provided valuable additional insights but were excluded to maximise participant privacy (Brännström et al., 2016; Giddens et al., 2022; Severn et al., 2012; Zimmer et al., 2022). Although useful for collecting data from a larger number of participants, the survey approach adopted limited the depth and richness of the qualitative data that could be obtained from participants and opportunities to explore their unique circumstances and the context informing their perspectives (Fossey et al., 2002). Response bias, the language of administration (English only) and the online nature of the administration may have played a role in determining the quality of the data collected and the response rate for the survey, and the sample itself was relatively small and may not have been sufficiently representative of the larger South African SLT&A population (Babbie & Mouton, 2001). The use of snowball and convenience sampling, although common and practical, may have introduced selection bias and limited the generalisability of the findings. Furthermore, the study design limited the ability to establish causality, and there are potential biases inherent in self-reported data.

## Conclusion

Despite these limitations, the findings from the study provide valuable insights into the professional quality of life of South African SLT&As as well as factors that they felt played an important role in determining the positive and negative emotional impact of their work. The qualitative data also revealed shared difficulties and specific ways in which South African SLT&As and SLT&As in similar contexts could be supported. This information may contribute to the development of future research trajectories and intervention strategies that reduce compassion fatigue and enhance satisfaction, retention and the capacity to offer effective and compassionate treatment for SLT&As in South Africa and internationally.
